# Strengthening a mental illness management questionnaire for clinical associates through expert validation and cognitive interviews

**DOI:** 10.4102/sajpsychiatry.29i0.1985

**Published:** 2023-02-28

**Authors:** Saiendhra V. Moodley, Jacqueline Wolvaardt, Christoffel Grobler

**Affiliations:** 1 School of Health Systems and Public Health, Faculty of Health Sciences, University of Pretoria, Pretoria, South Africa

## Background

The supply and distribution of specialist mental health professionals is a significant barrier to providing access to mental health services in South Africa (SA).^1,2^ Clinical associates are a clinically trained cadre that could be utilised in mental health service provision in underserved areas. There is a lack of data on clinical associates’ training in mental health, their knowledge and confidence to manage mental health conditions, attitudes towards mental illness, and the mental health services that they currently provide in SA. The authors developed a questionnaire to assist us to obtain this information. With the exception of the attitudes component of the questionnaire, which used the validated 16-item Mental Illness Clinicians’ Attitudes version 4 scale (MICA v4),^3^ the questions were developed by the authors. Expert validation and cognitive interview processes were used in the final stages of development of the questionnaire. The purpose of this scientific letter is to outline the expert validation and cognitive interview processes followed and to reflect on their value in developing a mental illness management questionnaire for clinical associates.

## Expert validation

### Process

A group of experts (three family physicians and three psychiatrists) were identified and invited to complete a content validation form via e-mail.^4^ The three family physicians were selected from two university family medicine departments involved in clinical associate undergraduate training. The three psychiatrists were selected from the public sector at three different levels of care. One of the psychiatrists was involved in a clinical associate undergraduate training programme. They were provided with an executive summary of the research project protocol and the scope of practice regulations of clinical associates.^5^ The knowledge, confidence, practice and interest items that used scales were included in the content validation form. The MICA v4 items were not included. The relevant items requiring expert validation were provided in shaded blocks on the validation form. The experts were asked not to answer any questions but rather consider the construct the authors were hoping to measure and rate the question for representativeness, clarity and relevance.^4^ All six experts returned their content validation forms. The quantitative data were aggregated from the six content validation forms and the qualitative responses of the experts were extracted. The responses were then reviewed by the authors with changes being made to the questionnaire where necessary.

### Outcomes

#### Knowledge items

In this section, clinical associates were expected to self-assess their knowledge of mental health conditions and presentations they are likely to encounter in practice. The experts were split as to the clarity of the question ‘Please rate your knowledge of the following:…’ with the conditions and presentations listed thereafter. Two experts indicated it was ‘somewhat clear’, two experts indicated it was ‘quite clear’ and two experts indicated it was ‘very clear’. To address this, an introductory statement to the question was added. All the experts thought the conditions and presentations included (schizophrenia, bipolar 1 disorder, substance use disorders, depressive disorders, anxiety disorders, post-traumatic stress disorder and suicide risk) were ‘quite relevant’ or ‘highly relevant’. The experts suggested a variety of conditions to be added to adequately address the construct including epilepsy, dementia, intellectual disability, personality disorders, acute stress disorder, adjustment disorder, neurocognitive disorders, attention-deficit hyperactivity disorder (ADHD) and other disorders of childhood. One expert raised the issue that patient presentation with the signs and symptoms of mental illness (prior to a diagnosis being made) had not been covered and another expert felt additional psychiatric emergencies needed to be included. As a result of the expert validation process, three additional conditions viz. acute stress disorder, ADHD and dementia were added. Dementia and ADHD were added as they were each mentioned by two experts. Acute stress disorder was only mentioned by one expert but the expert made a convincing case for its inclusion viz. that it is ‘very common and needs a firm handling by the generalist in communities, given the rates of exposure to trauma and violence encountered’. In addition, a separate question was included for mental health presentations with the aggressive patient and confused patient added to suicide risk.

Confidence items: This section required clinical associates to rate their confidence in performing six tasks (mental health history, mental health examination, mini-mental state examination [MMSE], physical examination, counselling a patient and counselling a patient’s family) for four specified conditions or presentations (suspected depression, suspected substance abuse, suspected schizophrenia and suicide risk). All the experts felt that the four conditions and presentations and five of the six tasks were ‘quite relevant’ or ‘highly relevant’. Two of the experts felt doing a MMSE was only ‘somewhat relevant’ with one expert noting that there may not be value in doing an MMSE on all patients. One expert suggested that the tasks should be listed independent of the suspected conditions as the clinical associate would need to differentiate prospectively based on their assessment. Some experts observed that certain common presentations (e.g. the aggressive patient) and tasks that clinical associates would be expected to perform, (e.g. ordering relevant investigations, sedating patients, completing forms for 72-h observation, and prescribing treatment) had been omitted. As a result of expert feedback, this section was restructured into three separate parts viz. confidence to carry out different aspects of an assessment for a person presenting with (undifferentiated) mental health symptoms, confidence in managing certain specified mental health presentations (suicide risk, a confused patient, an aggressive patient, and a patient suspected to be exposed to traumatic events) and confidence to prescribe treatment and provide counselling for specified conditions and presentations.

Practice items: This section asked clinical associates to indicate whether their current work involved performing specified tasks for four conditions or presentations (suspected depression, suspected substance abuse, suspected schizophrenia and suicide risk) with the responses being ‘never’, ‘sometimes’ and ‘often’. An expert suggested defining ‘sometimes’ and ‘often’ on the questionnaire would help and this change was made. The ordering of relevant investigations as one of the specified tasks was once again observed as omission as was sedation of an aggressive or violent patient and subsequently these tasks were included in the questionnaire. Doing a MMSE was removed as a task for all the specified conditions and presentations and included separately as ‘assessing the cognitive functioning of a patient with confusion using a suitable cognitive screening test (e.g. Mini-Mental State Exam)’.

Interest items: This section required clinical associates to indicate their interest in receiving further training as well as working in mental health. The issues noticed by the experts in this section were relatively minor. One of the experts suggested separating the training and work-related questions as they were measuring two different constructs and this change to the questionnaire was made. The omission of a postgraduate diploma as an example of advanced training in mental health was observed by one of the experts and was corrected.

## Cognitive interviews

### Process

Once the questionnaire had been updated following the expert validation process, cognitive interviews were conducted on an individual basis with qualified clinical associates involved in patient care to ensure that respondents interpret items as intended by the researchers.^4^ Cognitive interviews are useful as they allow one to assess how potential participants interpret questionnaire items and whether this interpretation aligns with what the researcher intended with each item.^4^ The cognitive interviews used a hybrid model consisting of the ‘think-aloud’ approach and some verbal probing.^6^ The initial plan was to interview 10 clinical associates but data saturation was reached after five interviews and no further interviews were conducted. The interviewer made notes on a blank questionnaire as the interviewee thought aloud for each question and responded to verbal probes. The interviews were audio-recorded in the event that any responses were missed, and the interviewer needed to refer back to the recording. The MICA v4 items^3^ did form part of the cognitive interview to flag any items that potentially could be misinterpreted by clinical associates but not with the intention to make any changes to the validated MICA v4 instrument.

### Outcomes

With respect to the socio-demographic characteristics and training sections of the questionnaire, a question regarding ‘current employment status’ was found to be unclear, missing some important categories (such as employed by an academic institution or self-employed) and did not account for clinical associates who may have had multiple employers. Changes were made to the questionnaire to address these issues. A question on work setting was also modified, with tertiary and central hospitals aggregated into a single category as interviewees were not clear on the difference between these two levels. In addition, ‘academic institution’ and ‘unemployed’ were added as categories for this question. In terms of items related to their training, the question ‘how long have you been practising as a clinical associate?’ was changed to ‘how long has it been since you qualified as a clinical associate?’ as it was pointed out by one of the interviewees that clinical associates may not necessarily have been practising as clinical associates in a clinical setting after qualifying.

With respect to the confidence and practice items, the task of ‘prescribing treatment’ to a patient needed to be clarified and this was changed to ‘prescribing pharmacological treatment’ in the final version of the questionnaire. With respect to the confidence items related to managing mental health presentations, one of the interviews picked up that the incorrect Likert scale (very poor-excellent rather than not at all confident-very confident) was being used, which was corrected in the final questionnaire. For the practice items, the term ‘current job’ was changed to ‘current work’ to account for clinical associates who may have more than one job.

Two of the MICA v4 items^3^ ‘People with a severe mental illness are dangerous more often than not’ and ‘Health/social care staff know more about the lives of people treated for a mental illness than do family members or friends’ were flagged as the wording was found to be confusing.

## Conclusion

The expert validation process resulted in several significant changes and the restructuring of parts of the questionnaire. The cognitive interview process resulted in clarification of some items and changes in the options that could be selected for a few questions. A straightforward and relatively quick expert validation and cognitive interview process led to significant improvements in a survey questionnaire to determine the knowledge, confidence, practices and interest related to the management of mental illness.
